# miR-214 ameliorates acute kidney injury via targeting DKK3 and activating of Wnt/β-catenin signaling pathway

**DOI:** 10.1186/s40659-018-0179-2

**Published:** 2018-09-04

**Authors:** Xiaoguang Zhu, Wenwen Li, Huicong Li

**Affiliations:** 1 Department of Nephrology, Huaihe Hospital of Henan University, No. 8, Baobei Road, Gulou District, Kaifeng, 475000 China; 2 Department of Cardiology, Huaihe Hospital of Henan University, Kaifeng, 475000 China

**Keywords:** miR-214, Acute kidney injury, *Dkk3*, Wnt/β-catenin signaling pathway

## Abstract

**Background:**

miR-214 was demonstrated to be upregulated in models of renal disease and promoted fibrosis in renal injury independent of TGF-β signaling in vivo. However, the detailed role of miR-214 in acute kidney injury (AKI) and its underlying mechanism are still largely unknown.

**Methods:**

In this study, an I/R-induced rat AKI model and a hypoxia-induced NRK-52E cell model were used to study AKI. The concentrations of kidney injury markers serum creatinine, blood urea nitrogen, and kidney injury molecule-1 were measured. The expressions of miR-214, tumor necrosis factor-α, interleukin (IL)-1β, IL-6, were detected by RT-qPCR. The protein levels of Bcl-2, Bax, Dickkopf-related protein 3, β-catenin, c-myc, and cyclinD1 were determined by western blot. Cell apoptosis and caspase 3 activity were evaluated by flow cytometry analysis and caspase 3 activity assay, respectively. Luciferase reporter assay was used to confirm the interaction between miR-214 and *Dkk3*.

**Results:**

miR-214 expression was induced in ischemia–reperfusion (I/R)-induced AKI rat and hypoxic incubation of NRK-52E cells. Overexpression of miR-214 alleviated hypoxia-induced NRK-52E cell apoptosis while inhibition of miR-214 expression exerted the opposite effect. *Dkk3* was identified as a target of miR-214. Anti-miR-214 abolished the inhibitory effects of DKK3 knockdown on hypoxia-induced NRK-52E cell apoptosis by inactivation of Wnt/β-catenin signaling. Moreover, miR-214 ameliorated AKI in vivo by inhibiting apoptosis and fibrosis through targeting *Dkk3* and activating Wnt/β-catenin pathway.

**Conclusion:**

miR-214 ameliorates AKI by inhibiting apoptosis through targeting *Dkk3* and activating Wnt/β-catenin signaling pathway, offering the possibility of miR-214 in the therapy of ischemic AKI.

**Electronic supplementary material:**

The online version of this article (10.1186/s40659-018-0179-2) contains supplementary material, which is available to authorized users.

## Background

Acute kidney injury (AKI), one of the most common complications of major surgical operations and sepsis accompanied by loss of kidney function, can be evaluated through monitoring the changes of renal function markers, such as serum creatinine (SCr), and blood urea nitrogen (BUN) levels [1, 2]. AKI is associated with higher incidence of mortality and morbidity, prolonged hospital stay, and accelerated chronic kidney disease (CKD) or end-stage renal disease (ESRD) [3, 4]. Renal ischemia–reperfusion (I/R) injury has been demonstrated as the major cause of AKI [5]. The pathophysiological process of AKI is widely known to encompass acute tubular epithelial cell damage, excessive inflammation, acute vascular dysfunction, and fibrosis in response to I/R injury [6]. Although several clinical symptomatic supportive treatments are available, there are still no effective therapies for the treatment of AKI [7].

microRNAs (miRNAs), a class of endogenous, small non-coding RNA molecules with 19–25 nucleotides in length, has emerged as important regulators of gene expression. miRNAs are reported to be involved in a variety of kidney diseases via regulating cell proliferation, apoptosis, differentiation, and development [8]. A growing body of evidence has highlighted the importance of miRNAs in the pathogenesis of AKI, facilitating the development of diagnostic and therapeutic strategies of AKI [9, 10]. Previous studies discovered that miR-214 was up-regulated in models of renal disease and promoted fibrosis in renal injury independent of TGF-β signaling in vivo [11, 12]. However, the detailed roles of miR-214 in AKI and its underlying mechanisms are still largely unknown.

Wnt/β-catenin signaling pathway, an evolutionarily conserved and key developmental signaling cascade, is implicated in the regulation of various biological processes, including AKI and diabetic nephropathy (DN) [13, 14]. Wnt/β-catenin pathway is reactivated after kidney injury to participate in the development and progression of renal fibrotic lesions in many animal models and human kidney disorders [15, 16]. Emerging evidence has shown that Wnt/β-catenin signaling plays a pivotal role in promoting kidney repair and regeneration after AKI induced by either I/R or nephrotoxins [17]. Dickkopf (DKK) proteins, an evolutionarily conserved family consisting of DKK1, 2, 3, and 4, have been reported to function as regulators of Wnt/β-catenin pathway [18]. They encode secreted proteins that typically antagonize Wnt/β-catenin signaling, by inhibiting the Wnt coreceptors LRP5 and 6 [19]. A previous study revealed that Dkk3 could interact with Kremen and LRP with comparable binding energies and block Wnt signaling [20]. microRNA-214 has been uncovered to negatively regulate the WNT/β-catenin signaling in human breast cancer and hepatocellular carcinoma [21, 22]. This study was designed to investigate the functions and mechanisms of miR-214 in I/R-induced AKI rat model and hypoxia-induced NRK-52E cell model.

## Methods

### Animals

All animal protocols were performed in accordance with the guidelines for the Care and Use of Laboratory Animals and approved by the Ethics Committee of Henan University of Huaihe Hospital. Healthy male Sprague–Dawley rats (275–300 g weight, and 11–12 weeks) were obtained from the Beijing Vital River Laboratory Animal Center. Rats were housed under standardized conditions with a constant temperature (23–24 °C), 50% humidity, and a light/dark cycle of 12:12 h, and allowed free access to food and water before use in experiments.

### Construction of rat AKI model

The rat AKI model was constructed by renal I/R surgery, as described previously [23]. Briefly, rats were anesthetized by intraperitoneal injection of pentobarbital sodium (50 mg/kg; Sigma-Aldrich) and body temperature of rats were maintained at 37 °C using heating clamps during surgery. After rats underwent an abdominal incision, bilateral renal pedicles were occluded with a microvascular clamp for 30 min to induce renal ischemia. The clamps were removed for reperfusion, and then the abdomen was closed in two layers with sutures. For the sham group, rats were subjected to the same surgical procedure, except the renal pedicles were not clamped, and rats without I/R surgery were used as controls. The rats in rat AKI model group and sham group were then administered intraperitoneally with 10 mg/kg miR-214 mimic (miR-214) or miRNA negative control (miR-con) (GenePharma Co. Ltd, Shanghai, China) in saline. After reperfusion for 12, 48 and 72 h, the rats were euthanized and then blood, urine, and kidney tissues were collected.

### Renal function measurement

Serum creatinine and blood urea nitrogen (BUN) in the serum of blood samples were measured using a creatinine assay kit (BioAssay Systems, Hayward, CA) and a Hitachi 7060 automatic biochemistry analyzer (Hitachi, Tokyo, Japan), respectively. The urine concentration of kidney injury molecule-1 (Kim-1) was measured by a commercially available ELISA Kit (Cosmo Bio, Tokyo, Japan).

### Cell culture and transfection

A rat renal proximal tubular cell line (NRK-52E cells) was purchased from American Type Culture Collection (ATCC, Manassas, VA, USA). NRK-52E cells were cultured in Dulbecco’s modified Eagle’s medium (DMEM; Sigma-Aldrich, St. Louis, MO, USA) containing 10% fetal bovine serum (FBS; Gibco, Carlsbad, CA, USA), 0.15% sodium bicarbonate (Sigma-Aldrich), 4 mM l-glutamine (Sigma-Aldrich), and 1% streptomycin/penicillin (Invitrogen, CA, Carlsbad, USA) in a humidified atmosphere chamber containing 5% CO_2_ at 37 °C. For hypoxia experiments, cells were incubated in serum-free medium for 24 h in a hypoxic chamber (AnaeroPack; Mitsubishi Gas Chemical Co., Inc., Tokyo, Japan) containing 1% O_2_, followed by reoxygenation for a further 24 h to generate hypoxia/reoxygenation (H/R) cell models. The control group was exposed to normoxic conditions for 48 h.

miR-214 mimic, mimic control (miR-con), miR-214 inhibitor (anti-miR-214), inhibitor control (anti-miR-con), siRNA against *Dkk3* (si-*Dkk3*), and siRNA control (si-con) were obtained from GenePharma Co. These oligonucleotides were transfected into NRK-52E cells using Lipofectamine 2000 (Invitrogen).

### Determination of caspase 3 activity

After NRK-52E cells were collected and lysed, and caspase 3 activity was measured using Caspase-3 Colorimetric Activity Assay Kit (Millipore, Billerica, MA, USA) according to kit instructions. The absorbance at 405 nm was measured using an ELISA reader (Bio-Rad Laboratories, Inc., Hercules, CA, USA).

### Cell apoptosis assay

Cell apoptosis was analyzed by flow cytometry following the annexin V-fluorescein isothiocyanate (FITC) Apoptosis Detection Kit II (BD Biosciences, San Jose, CA, USA) and propidium iodide (PI) staining according to the manufacturer’s instructions. Briefly, cells were collected, washed twice with cold phosphate-buffered saline (PBS), and resuspended in binding buffer. 100 μL of cell suspension (2 × 10^5^ cells) was transferred to a 96-well plate, and incubated with 10 μL of Annexin V-FITC and 5 μL of propidium iodide (PI) for 15 min at room temperature in the dark. The apoptotic cells, including FITC-positive and PI-negative cells (early apoptotic), were detected with a FACS flow cytometer (BD Biosciences).

### Luciferase reporter assay

Emerging evidence suggests that Wnt/β-catenin signaling plays a pivotal role in promoting kidney repair and regeneration after AKI induced by either IR or nephrotoxins [18]. Therefore, the underlying targets, which could regulate Wnt/β-catenin signaling, were predicted by Targetscan. Dkk3, an inhibitor of Wnt/β-catenin signaling, was selected and investigated in the study. Wild-type or seed-region mutated *Dkk3* 3′UTR sequences were synthesized and cloned into pmirGLO dual-luciferase miRNA target expression vectors (Promega, Madison, WI, USA), namely *Dkk3*-WT and *Dkk3*-MUT. NRK-52E cells were seeded into 24-well plates at a density of 1.0 × 10^5^/well and cotransfected with miR-214 or miR-con, and *Dkk3*-WT or DKK3-MUT, together with pRL-TK renilla plasmids using Lipofectamine 2000 (Invitrogen). At 48 h posttransfection, cells were harvested for measuring the luciferase activities with the Dual-Luciferase Assay System (Promega). Renilla luciferase activities were detected as a normalization.

### Reverse transcription-quantitative polymerase chain reaction (RT-qPCR)

Total RNA was extracted from resected tissues and cells using TRIzol^®^ reagent (Invitrogen). For the detection of miR-214 expression, 1 μg of total RNA was reversely transcribed into cDNA using Taqman™ microRNA reverse transcription kit (Life Technologies, Gaithersburg, MD, USA) and RT-PCR was performed using TaqMan™ MicroRNA Assay kit (Applied Biosystems, Foster City, CA, USA). For the measurement of *Dkk3* mRNA expression, the first-strand cDNA was synthesized from 1 μg of total RNA by a High-Capacity cDNA Archive Kit (Applied Biosystems) and RT-PCR was conducted using a SYBR Premix Ex Taq II Kit (Applied Biosystems). qRT-PCR reaction was carried out using a 7500 Fast Real-Time Sequence detection system (Applied Biosystems). The relative gene expression of miR-214 and *Dkk3* was calculated using the 2^−ΔΔCt^ method. GAPDH and U6 were used as the internal controls for miR-214 and *Dkk3*, respectively.

### Western blot

Cells were harvested and lysed using ice-cold radioimmunoprecipitation assay (RIPA) lysis buffer (Beyotime, Jiangsu, China). Protein concentration was quantified using a Bicinchoninic Acid Protein Assay kit (Sangon Biotech Co., Ltd., Shanghai, China). Total of 10 μg protein were subjected to 10% SDS-PAGE and then transferred onto PVDF membranes (Millipore). The membranes were blocked with 5% non-fat milk for 1 h at room temperature and then incubated with primary antibodies fibronectin (BD Biosciences), Bax, Bcl-2, DKK3, β-catenin, c-myc, cyclin D1 and β-actin (Cell Signaling Technology, Beverly, MA, USA), followed by further incubation with HRP-labeled secondary antibody (Abcam, Cambridge, MA, USA) for 2 h. The protein signals were visualized using an enhanced chemiluminescence system (ECL™; Amersham, Little Chalfont, UK).

### Statistical analysis

All experimental data were presented as mean ± standard deviation (SD). Statistical analyses were performed using SPSS software (version 19.0, IBM, Chicago, IL, USA). Statistical differences between different groups were evaluated by one-way analysis of variance (ANOVA) with Student–Newman–Keuls (SNK) post hoc test and Student’s *t*-test. Differences were considered to be statistically significant when *P* values < 0.05.

## Results

### Establishment of I/R-induced AKI rat model and hypoxia-induced AKI cell model

**Fig. 1:**
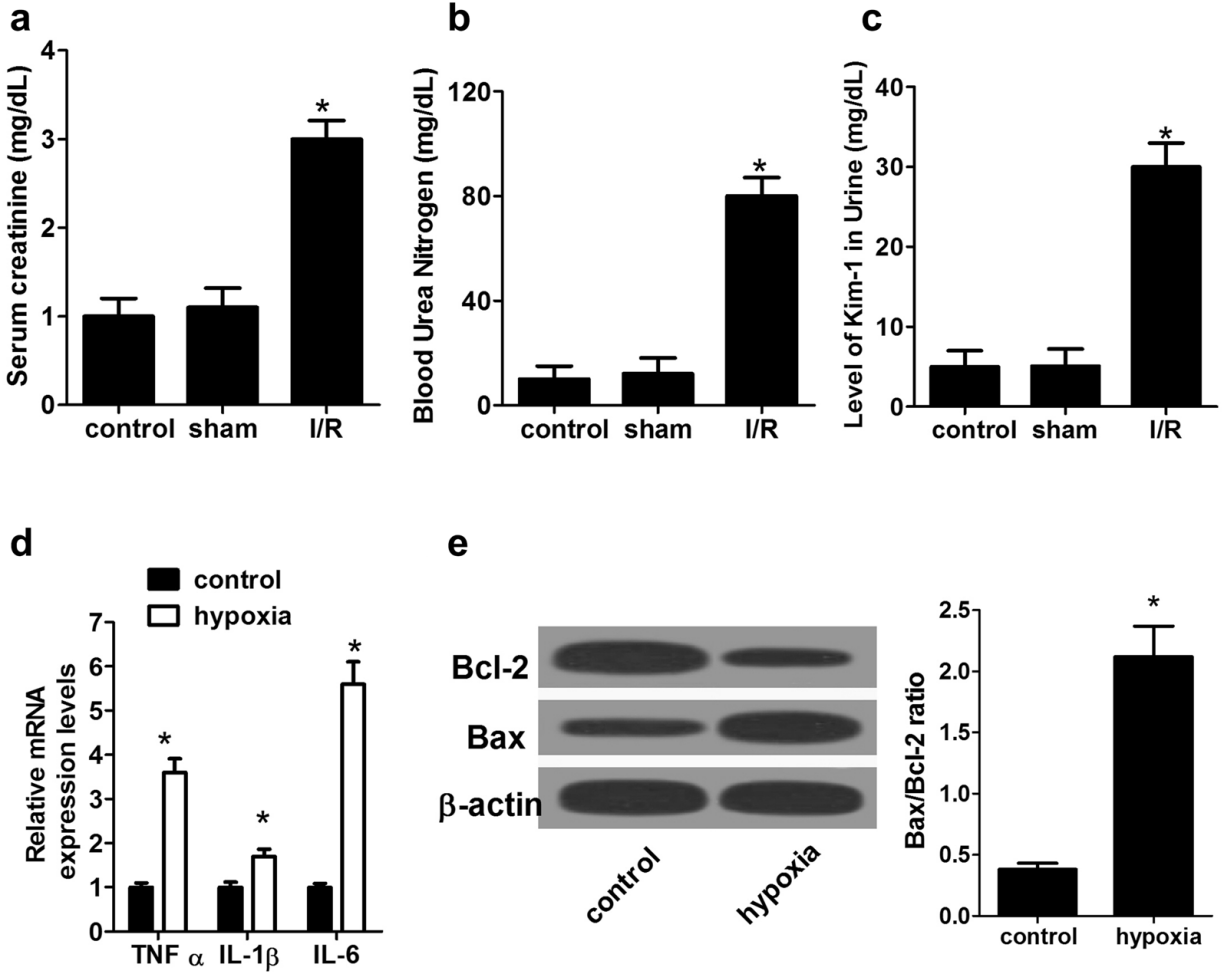
Evaluation of rat AKI model following I/R surgery and NRK-52E cell model following hypoxia treatment. The serum levels of SCr (**a**), BUN (**b**), and urine Kim-1 (**c**) in I/R-induced rat AKI models at 24 h after surgery were measured. **d** The mRNA expressions of TNF-α, IL-1β, and IL-6 in hypoxia-induced NRK-52E cell I/R model were detected by qRT-PCR. **e** The protein levels of Bcl-2 and Bax in hypoxia-induced NRK-52E cell I/R models were assessed by western blot. *n* = 6 rats/group. *P < 0.05

I/R-induced AKI rat model and hypoxia-induced AKI cell model were established to explore the role of miR-214 in AKI progression. The success of I/R-induced rat AKI model was firstly evaluated by measuring the serum levels of SCr, BUN, and urine Kim-1, a potential biomarker in ischemic AKI [24], at 24 h after surgery. As compared with sham group, the serum levels of SCr (Fig. 1a), BUN (Fig. 1b), and urine Kim-1 (Fig. 1c) were all significantly increased in I/R-induced rat AKI models. Since inflammation and tubular epithelial cell apoptosis are the major features of AKI [25], the effectiveness of hypoxia-induced NRK-52E cell model was further assessed by detecting the expression of inflammatory factors including tumor necrosis factor (TNF)-α, interleukin (IL)-1β, and IL-6, and examining apoptosis [26]. qRT-PCR results demonstrated that mRNA expressions of TNF-α, IL-1β, and IL-6 were markedly promoted under hypoxia compared with cells under normoxic conditions (Fig. 1d). Also, western blot analyses demonstrated that hypoxia-induced NRK-52E cells showed an obvious decrease of Bcl-2 protein level and an apparent increase of Bax level (Fig. 1e), suggesting the emergence of apoptosis. All these results indicated the successful construction of I/R-induced rat AKI models and hypoxia-induced cell I/R model.

### Overexpression of miR-214 alleviated hypoxia-induced apoptosis in NRK-52E cells

**Fig. 2:**
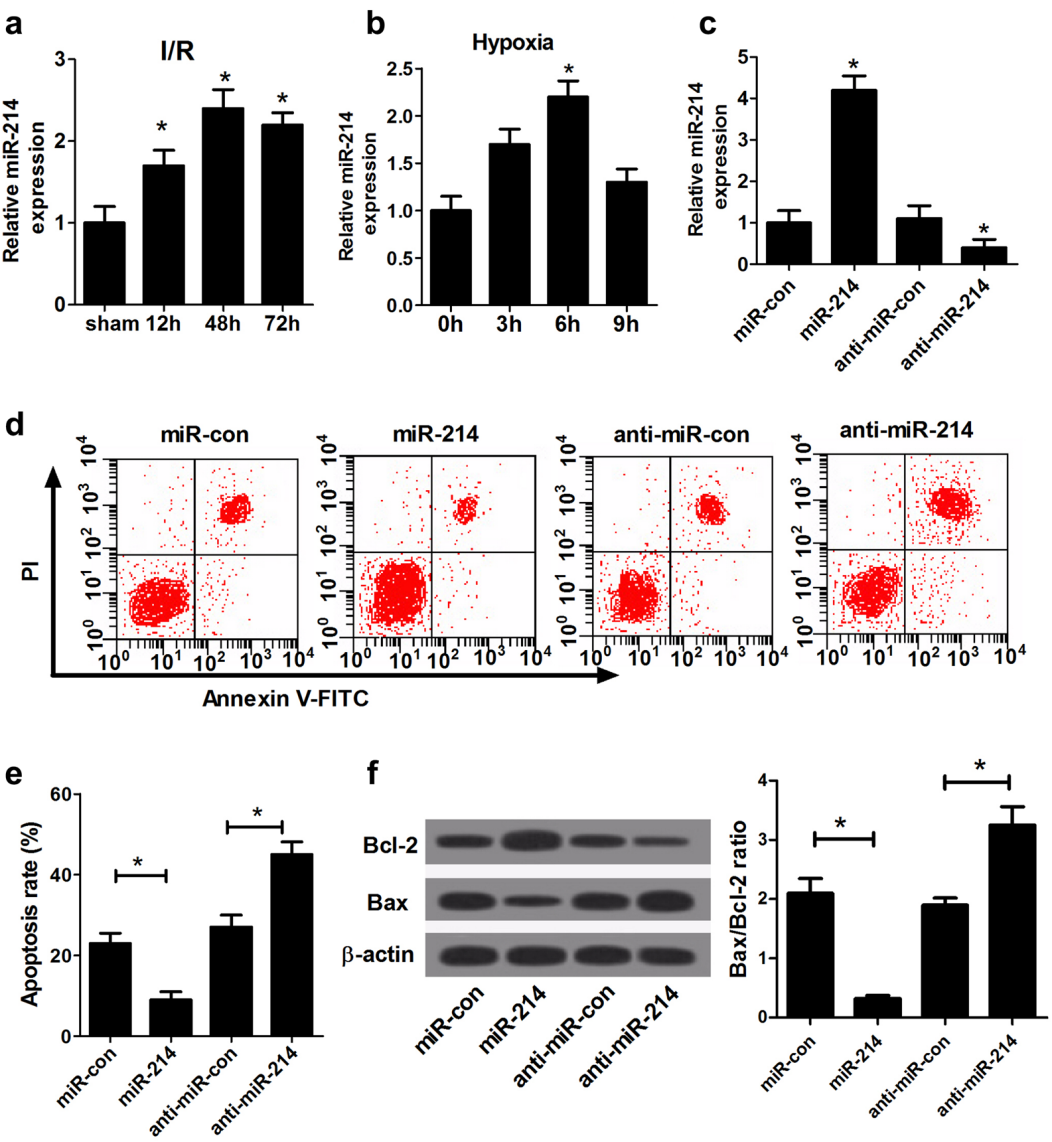
miR-214 attenuated NRK-52E cell apoptosis under hypoxia. **a** qRT-PCR analysis of miR-214 expression in kidney cortex of I/R-induced rat AKI models after 12, 48 and 72 h of reperfusion. *n* = 6 rats/group. **b** qRT-PCR analysis of miR-214 expression at 0, 3 h, 6 h, and 9 h during hypoxic incubation of cultured NRK-52E cells. **c** qRT-PCR analysis of miR-214 expression in NRK-52E cells after transfection with miR-214, anti-miR-214, or matched controls. **d–e** Flow cytometry was used to analyze the apoptotic rate of NRK-52E cells after transfection with miR-214, anti-miR-214, or respective controls during hypoxia incubation. **f** Western blot was performed to detect the protein levels of Bcl-2 and Bax in NRK-52E cells after introduction with miR-214, anti-miR-214, or corresponding controls under hypoxia. Each experiment was independently repeated 3 times. *P < 0.05

The expression of miR-214 in kidney tissues of I/R-induced rat AKI models was analyzed by qRT-PCR and the in vivo results demonstrated that miR-214 expression was aberrantly upregulated in I/R-induced rat AKI models after 12, 48 and 72 h of reperfusion compared to that in sham group (Fig. 2a). The expression of miR-214 during hypoxic incubation of cultured NRK-52E cells was also evaluated by qRT-PCR and the in vitro results demonstrated that miR-214 expression was increased at 0–6 h, and reached the highest at 6 h after hypoxia treatment (Fig. 2b). These results indicated that miR-214 was induced during renal I/R in kidney tissues and hypoxia in cultured renal proximal tubular cells. To determine the role of miR-214 in AKI, loss- and gain-of-function experiments were performed in hypoxia-induced NRK-52E cells by transfecting with miR-214 or anti-miR-214. qRT-PCR results demonstrated that miR-214 transfection dramatically promoted miR-214 expression and introduction of anti-miR-214 significantly lowered miR-214 expression (Fig. 2c). Flow cytometry analysis showed that exogenous expression of miR-214 led to an obvious suppression on hypoxia-induced apoptosis in NRK-52E cells, while miR-214 inhibitor resulted in a marked enhancement of apoptotic rate in NRK-52E cells during hypoxia treatment (Fig. 2d, e). Meanwhile, western blot results indicated that forced expression of miR-214 promoted Bcl-2 level and repressed Bax level, while down-regulation of miR-214 elicited the opposite effects on Bcl-2 and Bax expression (Fig. 2f). Therefore, these results suggested that miR-214 induced in I/R may exert a protective effect on tubular cell injury by inhibiting apoptosis.

### miR-214 suppressed DKK3 expression in NRK-52E cells

**Fig. 3:**
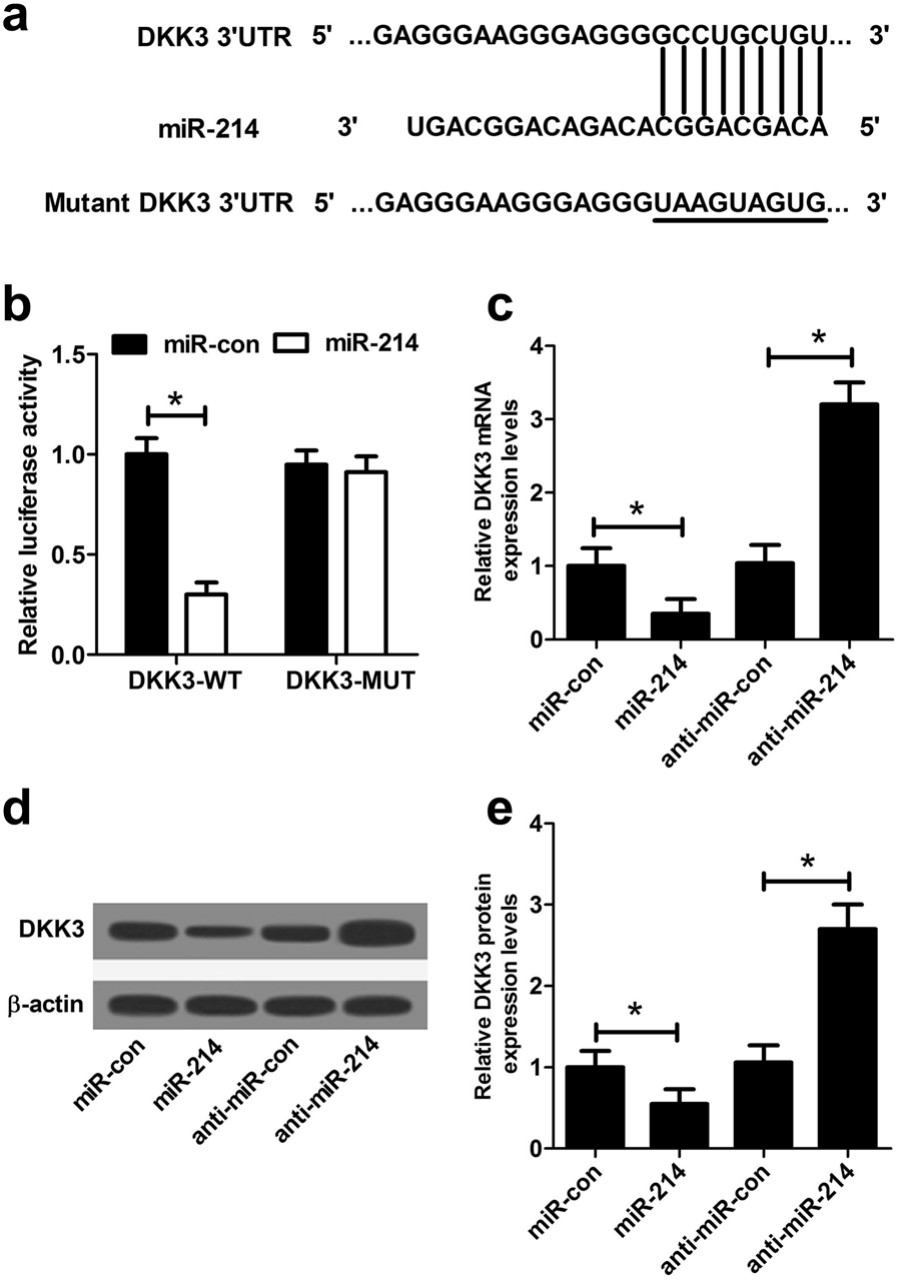
*Dkk3* was a target of miR-214 in NRK-52E cells. **a** The predictive wild-type miR-214 binding sites in the 3′UTR of *Dkk3* and the corresponding mutant binding sites were displayed. **b** The relative luciferase activity was measured by luciferase reporter assay after NRK-52E cells were cotransfected with *Dkk3*-WT or *Dkk3*-MUT and miR-214 or miR-con. The mRNA (**c**) and protein (**d**, **e**) levels of DKK3 were examined by qRT-PCR and western blot in NRK-52E cells introduced with miR-214, anti-miR-214, or matched controls. Each experiment was independently repeated 3 times. *P < 0.05

Bioinformatics analyses predicted that 3′UTR of *Dkk3* contained some conserved binding sequences of miR-214 (Fig. 3a). To confirm whether *Dkk3* was a target of miR-214 in NRK-52E cells, either wild-type or mutated 3′UTR sequences of *Dkk3* were cloned into luciferase reporter plasmids. Luciferase reporter assay results proved that overexpression of miR-214 significantly restrained the relative luciferase activity of DKK3-WT, but exerted no inhibitory effect on the relative luciferase activity of *Dkk3*-MUT (Fig. 3b). Additionally, endogenous DKK3 expression at mRNA and protein levels in NRK-52E cells transfected with miR-214 or anti-miR-214 were evaluated by qRT-PCR and western blot. As displayed in Fig. 3c–e, DKK3 expression at both mRNA and protein levels was prominently reduced following ectopic expression of miR-214 and enhanced by miR-214 inhibitor. Together, these data revealed that miR-214 targeted *Dkk3* by binding to the 3′UTR of *Dkk3* in NRK-52E cells.

### Anti-miR-214 abolished the inhibitory effects of DKK3 knockdown on hypoxia-induced apoptosis in NRK-52E cells by inactivation of Wnt/β-catenin signaling

**Fig. 4:**
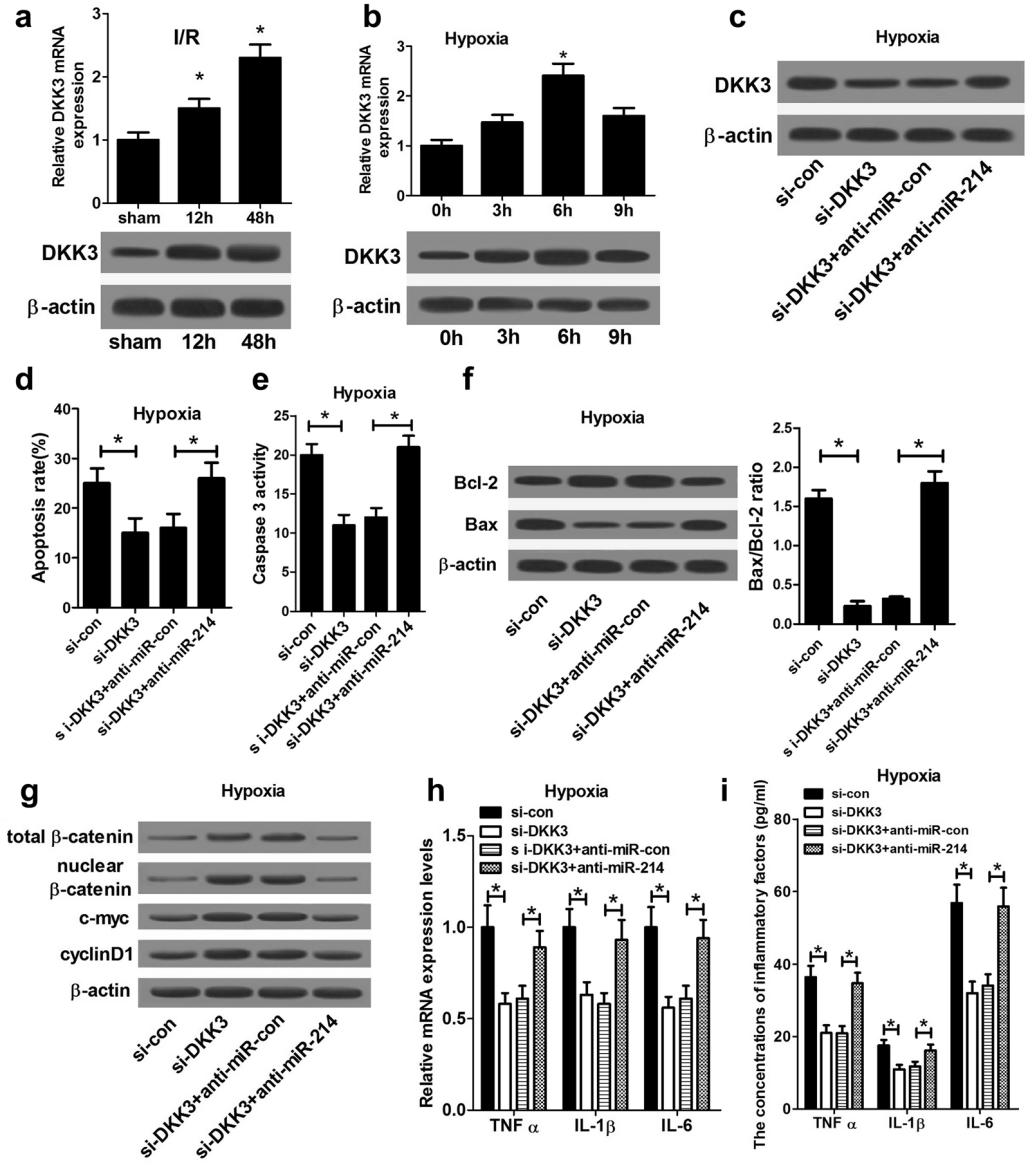
Anti-miR-214 abolished the inhibitory effect of DKK3 knockdown on hypoxia-induced apoptosis in NRK-52E cells by inactivation of Wnt/β-catenin pathway. Western blot analysis of DKK3 level in kidney tissues of I/R-induced rat AKI models after 30 min of ischemia and 12 or 48 h of reperfusion (**a**) and cultured NRK-52E cells after hypoxia treatment (**b**). NRK-52E cells transfected with either si-DKK3 alone or combined with anti-miR-214 were incubated under hypoxia, followed by detection of DDK3 protein expression (**c**), apoptosis (**d**), caspase 3 activity (**e**), Bcl-2 and Bax protein levels (**f**), β-catenin, c-myc, cyclin D1 expression (**g**) mRNA expressions of TNF-α, IL-1β and IL-6 (**h**), and the concentrations of TNF-α, IL-1β and IL-6 (**i**). Each experiment was independently repeated 3 times. *P < 0.05

Firstly, the protein expression of DKK3 was analyzed in I/R-induced rat AKI models and hypoxia-induced NRK-52E cell model. The results displayed that the protein level of DKK3 in kidney tissues of I/R-induced rat AKI models was increased after 30 min of ischemia and 12 or 48 h of reperfusion when compared with sham group (Fig. 4a). Moreover, *Dkk3* expression was also enhanced in NRK-52E cells after hypoxia treatment, and the increase was most significant at 6 h after hypoxic treatment (Fig. 4b). To explore the role of DKK3 in miR-214-mediated renal protection, NRK-52E cells were transfected with si-DKK3 alone or along with anti-miR-214. As expected, DKK3 level was markedly reduced by introduction of si-DKK3, but DKK3 expression was notably restored by cotransfection with anti-miR-214 (Fig. 4c). Flow cytometry analyses demonstrated that DKK3 knockdown significantly blocked hypoxia-induced apoptosis in NRK-52E cells, while this inhibitory effect was weakened after down-regulating miR-214 (Fig. 4d). Similarly, caspase 3 activity assay revealed that caspase 3 activity was strikingly reduced by DKK3 knockdown, however, inhibition of miR-214 relieved si-DKK3-induced decrease of caspase 3 activity in NRK-52E cells following hypoxia treatment (Fig. 4e). In line with the above results, DKK3 knockdown markedly increased Bcl-2 expression and decreased Bax level, which was substantially abrogated by miR-214 repression (Fig. 4f). DKK3 has been reported to function as a negative regulator of Wnt/β-catenin pathway. Thus, the effects of DKK3 knockdown and miR-214 inhibition on Wnt/β-catenin pathway were analyzed by detecting the protein levels of β-catenin and its downstream genes (c-myc, and cyclin D1). As shown in Fig. 4g, the protein levels of β-catenin, c-myc, and cyclin D1 were significantly increased in NRK-52E cells after transfection with si-DKK3 compared with si-NC group (Fig. 4g). However, cotransfection with si-DKK3 and anti-miR-214 significantly abated the increase of the protein levels of β-catenin, c-myc, and cyclin D1 induced by si-DKK3, suggesting that miR-214 suppression abolished DKK3 knockdown-induced inhibition on the Wnt/β-catenin pathway. Moreover, the mRNA expressions and concentrations of TNF-α, IL-1β, and IL-6 were significantly decreased in NRK-52E cells after transfection with si-DKK3 compared with si-NC group (Fig. 4h, i). Together, it is concluded that DKK3 knockdown could protect against tubular cell injury by blocking Wnt/β-catenin pathway, and this effect was mediated by miR-214.

### miR-214 ameliorated AKI in vivo by inhibiting apoptosis and fibrosis via targeting Dkk3 and activating Wnt/β-catenin pathway

**Fig. 5:**
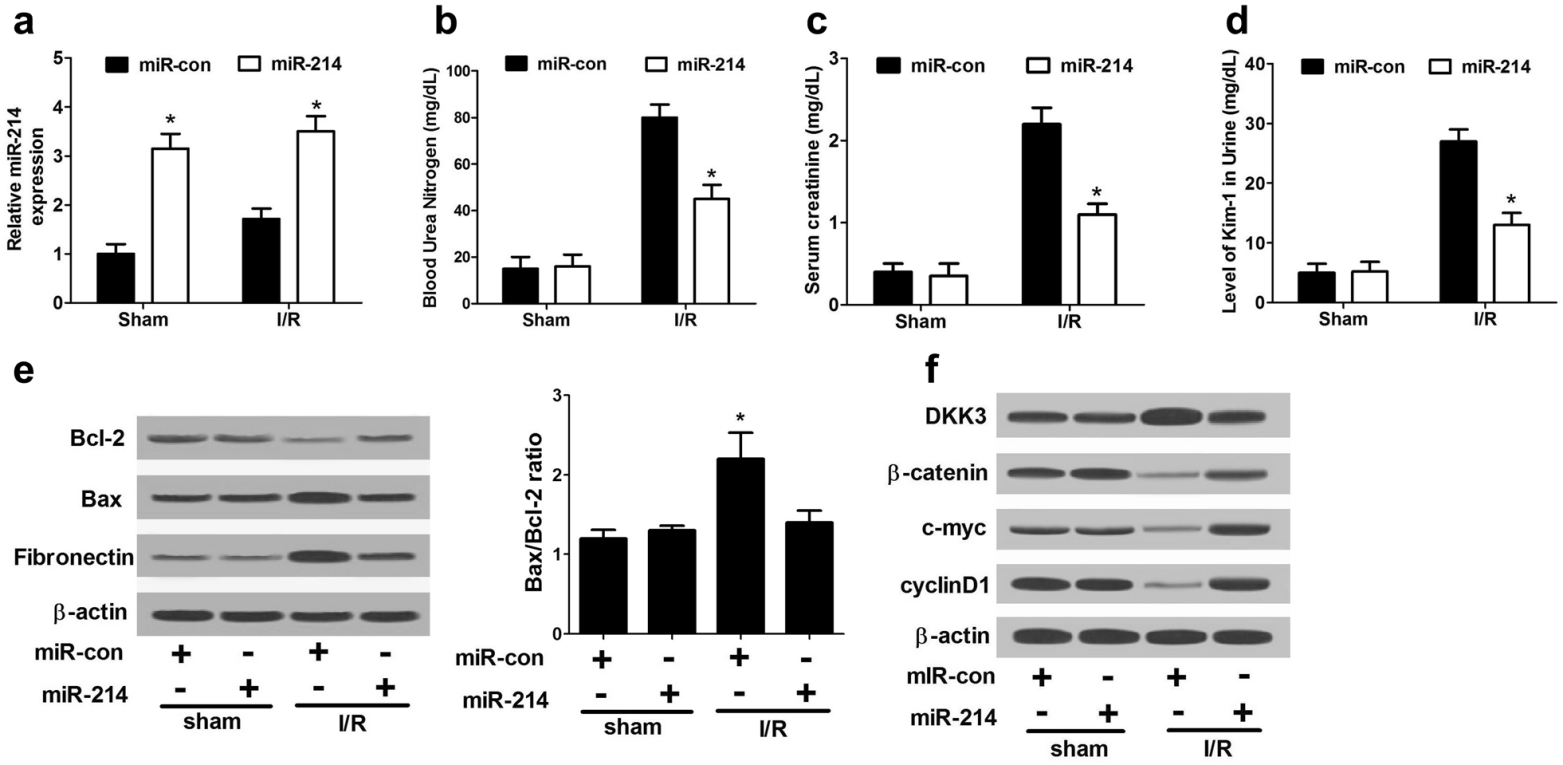
miR-214 protected against AKI in vivo through targeting *Dkk3* and activating Wnt/β-catenin pathway. miR-214 or miR-con was intraperitoneally injected into mice, followed by ischemic surgery. **a** qRT-PCR analysis of miR-214 expression in I/R-induced rat AKI model and sham mice. The serum levels of BUN (**b**), SCr (**c**), and Urine Kim-1 (**d**) level in I/R-induced rat AKI model and sham mice. **e** Western blot analysis of Bcl-2, Bax and fibronectin in I/R-induced rat AKI model and sham group. **f** The protein levels of DKK3, β-catenin, c-myc, and cyclin D1 in I/R-induced rat AKI model and sham group. Each experiment was independently repeated 3 times. *P < 0.05

To confirm the molecular mechanism of miR-214 involved in the pathogenesis of AKI in vivo, miR-214 or miR-con was intraperitoneally injected into mice, followed by ischemic surgery. Then the renal function was assessed through monitoring the serum levels of SCr and BUN, and urine Kim-1. qRT-PCR showed that miR-214 expression was significantly improved by miR-214 injection in I/R-induced AKI rats versus that in sham group (Fig. 5a). As compared with sham group, the concentrations of SCr (Fig. 5b), BUN (Fig. 5c), and urine Kim-1 (Fig. 5d) were all reduced following miR-214 treatment in I/R-induced AKI rats, suggesting the amelioration of AKI by miR-214. In addition, forced expression of miR-214 enhanced Bcl-2 level, reduced Bax level and decreased fibronectin protein level in I/R-induced rat AKI model relative to that in sham group (Fig. 5e), suggesting that overexpression of miR-214 inhibited apoptosis and fibrosis in I/R-induced rat AKI model. Furthermore, western blot analysis demonstrated that exogenous expression of miR-214 inhibited the protein level of DKK3 and promoted β-catenin, c-myc, and cyclin D1 levels in I/R-induced rat AKI model when compared with that in sham group (Fig. 5f), suggesting that miR-214 activated the Wnt/β-catenin pathway by targeting *Dkk3*. These data demonstrated that miR-214 ameliorated AKI in vivo by repressing apoptosis through targeting *Dkk3* and activating Wnt/β-catenin pathway.

## Discussion

In the present study, the I/R-induced rat AKI model and hypoxia-induced NRK-52E cell model were established to explore the significance of miR-214 in the development of AKI and its underlying mechanisms. It is found that miR-214 was induced in I/R-induced AKI rats and hypoxia-induced renal proximal tubular cells, and overexpression of miR-214 alleviated hypoxia-induced NRK-52E cell apoptosis. *Dkk3* was identified as a target of miR-214 in NRK-52E cells. Moreover, down-regulation of miR-214 abated the inhibitory effects of DKK3 knockdown on hypoxia-induced NRK-52E cell apoptosis by inactivation of Wnt/β-catenin pathway. In vivo experiments further disclosed that miR-214 ameliorated AKI in vivo by suppressing apoptosis and fibrosis through targeting *Dkk3* and activating Wnt/β-catenin pathway.

Accumulating evidence elucidates that dysregulated miRNAs are implicated in the pathogenesis of kidney injury and renal fibrosis [27, 28]. For instance, transfer of miR-489 expression from human cord blood endothelial colony-forming cell-derived exosomes reduced ischemic kidney injury by targeting phosphatase and tensin homolog (PTEN) through activation of Akt pathway [29]. miR-107 induced TNF-α secretion by targeting Dual-specificity phosphatase 7(DUSP7) in endothelial cells, triggering tubular cell injury in septic AKI [30]. miR-16 transactivated by CCAAT enhancer binding protein beta (C/EBP-β) attenuated kidney function and increased kidney apoptosis after I/R injury [31]. Several studies have focused on comprehensive investigations into the biological functions of miR-214. miR-214 expression was up-regulated in monocytes of patients with chronic kidney disease [32]. Notably, previous documents illustrated that miR-214 protected cells from hypoxia/reoxygenation (H/R) induced damage, and attenuated I/R-induced cardiac myocardial injury and I/R-induced cardiac myocyte apoptosis by modulating PTEN expression through activation of PI3K/Akt pathway [33, 34]. In addition, miR-214 and miR-21 were consistently modulated during renal injury [11]. Therefore, the biological roles of miR-214 and miR-21 may be also consistent in renal injury. Up-regulation of miR-21 by ghrelin ameliorated I/R-induced AKI by inhibiting the inflammatory responses and renal tubular epithelial cell apoptosis via stimulating PI3K/Akt pathway [35]. Besides, miR-21 overexpression suppressed kidney cell apoptosis induced by sepsis in AKI by affecting PTEN/PI3K/AKT signaling pathway [36]. In the present study, the rat AKI model was successfully established as demonstrated by the increased serum levels of SCr, BUN, and urine Kim-1. NRK-52E cell I/R model was also constructed accompanied by the enhanced expressions of inflammatory factors and the emergence of apoptosis. Also, miR-214 expression was induced in ischemic AKI and hypoxic incubation of kidney cells, similar with increased miR-489 expression in renal tubular cells after ischemic AKI [37]. Loss- and gain-of-function experiments demonstrated that miR-214 alleviated hypoxia-induced NRK-52E cell apoptosis, indicating that miR-214 ameliorated AKI by inhibiting apoptosis.

Wnt/β-catenin pathway is transiently activated after AKI, and identified as a protective response to minimize cell damage by facilitating tubular repair and regeneration [18]. For instance, Wnt agonist improved renal regeneration and function while attenuated inflammation and oxidative stress in the kidneys after I/R [38]. Administration with erythropoietin improved the kidney against I/R injury with activation of Wnt/β-catenin pathway [39]. However, the exaggerated and sustained activation of Wnt/β-catenin pathway may contribute to the transition of AKI to chronic kidney disease (CKD) [40]. Through interacting with the different proteins of Wnt signaling cascade, DKK3, a Wnt/β-catenin signaling antagonist, has been demonstrated to be implicated in numerous biological processes, including embryonic development and tumorigenesis [41]. DKK3 was reported to suppress the Wnt/β-catenin signaling in proximal tubular epithelial cells (PTECs), therefore inducing tubular cell death in proteinuric nephropathy [42]. In nucleus, β-catenin could bind to TCF/LEF and stimulate related downstream genes, including c-myc and cyclinD1 [43]. To explore the molecular mechanism by which miR-214 inhibited hypoxia-induced NRK-52E cell apoptosis, the potential targets of miR-214 were predicted. Luciferase reporter assay, RT-qPCR and western blot analyses demonstrated that *Dkk3* was a target of miR-214 and miR-214 suppressed DKK3 expression, which was also induced in ischemic AKI and hypoxia-induced NRK-52E cells. Mechanistic analysis demonstrated that miR-214 inhibition reversed the suppressive effect of DKK3 knockdown on hypoxia-induced NRK-52E cell apoptosis by inactivation of Wnt/β-catenin signaling in vitro, while miR-214 ameliorated AKI in vivo through decreasing apoptosis by targeting DKK3 and activating Wnt/β-catenin pathway. Although the data suggest that overexpression of miR-214 may be protective immediately after I/R, the long-term consequences of miR-214 up-regulation remain unclear. Previous studies showed that persistent gain of miR-214 in tubules leads to pro-inflammatory and profibrotic phenotypes, which may contribute to chronic kidney disease after AKI. Optimal dosing and timing of delivery of miR-214 mimics after AKI should therefore be further investigated to mitigate such potential late adverse effects.

## Conclusion

In conclusion, this study demonstrated that miR-214 was induced during ischemic AKI and hypoxic incubation of kidney cells. Upon induction, miR-214 may target DKK3 to suppress apoptosis via activation of Wnt/β-catenin pathway during ischemic or hypoxic renal damage. Therefore, miR-214 may serve as a new therapeutic target for the prevention and treatment of AKI.

## Additional file

**Additional file 1.** The original data of western blot in this study.

## Abbreviations

AKI: acute kidney injury
Kim-1: kidney injury molecule-1
SCr: serum creatinine
BUN: blood urea nitrogen
TNF-α: tumor necrosis factor-α
ESRD: end-stage renal disease
CKD: chronic kidney disease
miRNAs: microRNAs
DN: diabetic nephropathy
FBS: fetal bovine serum
